# Yellow Fever in the Olden Time

**Published:** 1897-11

**Authors:** 


### YELLOW FEVER IN THE OLDEN TIME.

In a rare pamphlet* before us we learn that "New York sustained in sixteen years (from 1791 to 1807) thirteen attacks of yellow fever, causing the death of at least five thousand persons, and each time compelling the flight from their homes and occupation of many thousands of the population. And it is another interesting fact that, since the year 1807, New York has been visited by it but twice, viz , 1819 and 1822, and the latter visitation was on the opposite side of the city, against which no complaints of nuisances could be made; and the commencement of this period of exemption was, moreover, coeval with the enforcement of a law for the filling up of *these* slips and the general improvement of those ancient haunts, in which the opponents of its importation so clearly saw the domestic source of the disease." This begins the statment of the *domestic origin* theory, coupled with a sneer at Noah Webster in his work on "Pestilence" that during the Revolutionary War our country was not visited by yellow fever which "*he* [the italics are not ours] would doubtless attribute to an interposition of Providence, though he does not hold so in express language. He speaks of it simply as a 'striking fact' in the middle of his labored efforts to prove the source of nearly all epidemics to lie in domestic local circumstances in combination with meteoric influences and the appearance of comets. A more rational solution of the circumstance may, we think, be found in the fact that during the war nearly all foreign commerce was suspended." The next point is well put in a sharp sentence, to wit.: "Upon the borders of the city in the immediate vicinity of the shipping, while the neighborhoods of filth and nastiness were exempt, particularly 'the Collect' in Center Street."

Then, again, from this pamphlet we glean that not sooner than 1805 were infected vessels prohibited from coming within 300 yards of the Island of New York after being discharged of their cargoes. The supplementary law of 1806 more explicitly restricts the vessels from the West Indies and the Mississippi, arriving between June and October, to only four days' detention at quarantine, and prohibits intercourse between their crews and the city of New York, except under regulations of the health officer. These were the days of sailing vessels, with their prolonged voyages, dirty holds, cramped berths, huddled crews and damp cargoes. Thus there were now acknowledged to be some other transmitters of pestilence besides the individual, as well as the recognition of the law that the longer the quarantine the greater the danger. Another report by the same author, before the same body, read August 5, 1857, upon the

*A "History Chronological and Circumstantial of the Visitations of Yellow Fever at New York," by John H. Griscom, M D., Physician to the N. Y. Hospital; Fellow of the N. Y. Academy of Medicine; read before the Academy and printed by permission.

epidemic of the preceding year, refers to the etiological explanation of the New Orleans Sanitary Commission of 1853, that there is a miasmatic origin of the fever, intensified by both a high temperature and a high degree of humidity. Here is introduced the metaphoric "shears of fate," a harmless instrument when either blade is missing. To this our author replies that, if a law, it is certainly inoperative in New York City or any of its environments. His citations are the epidemics, inclusive of the one of 1819, in all of which the visitations had their location on the southeastern margin in the vicinity of the wharves of the East River. Besides these instances, Brooklyn, in 1809, had lost between thirty and forty lives in a well-defined area of about two hundred yards semidiameter, the center of which was a vessel from Havana, on board of which the first case occurred. The first American novelist, Brown, many years ago wrote a most interesting chapter on the adventures of his hero in the Philadelphia epidemic of 1799, the same in which Benjamin Rush won imperishable renown.

In 1822 the fever shifted its nidus to the western margin of the city, on the Hudson River, the "residence of a population aristocratic, at least in cleanliness and the elegancies of life." At that time *the domestic origin* theorists were forced to yield the field even to the surrender of the graveyard of Trinity Church. The recital of the episodes in the epidemic of 1856 is exceedingly interesting, and the name of a pioneer sanitarian, the late Dr. Elisha Harris, is given a merited prominence for the best of work. But as we have hardly room even for scant justice, we must abide with only summaries. We learn that the total number of cases during the summer and autumn of 1856 was 538, more than one-third of whom died of "black vomit." The whole number of cases which occurred within a circle of five miles radius, having its center at the Marine Hospital, counted up to a figure not much below an additional six hundred. After the due crediting of the rigid quarantine laws, much of evil is attributed to the prevalence of winds sweeping along the lines of the pestilence and the function of the tides in floating the debris of ship refuse to the shores with their zigzag docks and reeking sewers.

Charleston, Norfolk, Baltimore, Philadelphia and Providence have their share of attention as pictures with grewsome details in this gallery of epidemiology, but enough has already been written in proof of Solomon's wisdom that "there is nothing new under the sun." We are also to bear in mind that this pamphlet adheres closely to the subject of preventive sanitation, and has naught to say of the Antilles as the cradle of the disease, or even of the northern portion of the west coast of Africa. Not so, however, of the sailor quarters, always so close to a harbor.

